# Comparative Study of Volatile Compounds and Expression of Related Genes in Fruit from Two Apple Cultivars during Different Developmental Stages

**DOI:** 10.3390/molecules26061553

**Published:** 2021-03-12

**Authors:** Shuaishuai Feng, Chengtai Yan, Tianhao Zhang, Miaomiao Ji, Ru Tao, Hua Gao

**Affiliations:** 1 State Key Laboratory of Crop Stress Biology in Arid Areas, College of Horticulture, Northwest A&F University, Yangling 712100, China; 18906480991@163.com (S.F.); yan18838916923@163.com (C.Y.); zth942579598@163.com (T.Z.); jmm18234472125@163.com (M.J.); m18191638821@163.com (R.T.); 2 Key Laboratory of Horticultural Plant Biology and Germplasm Innovation in Northwest China, Ministry of Agriculture, Northwest A&F University, Yangling 712100, China; 3 Pomology Institute, Zibo Academy of Agricultural Sciences, Zibo 255000, China

**Keywords:** apple cultivars, fruit development, fruit maturation, volatile compounds, esters, gene expression

## Abstract

Aromatic volatile compounds are important contributors to fruit quality that vary among different cultivars. Herein, headspace solid-phase microextraction coupled with gas chromatography-mass spectrometry was used to determine changes in volatile compounds and related gene expression patterns in “Ruixue” and “Fuji” apples (*Malus domestica* Borkh.) during fruit development and maturation. Volatile compounds detected in the fruit of both cultivars exhibited similar trends across different developmental stages. In the early stages of “Ruixue” fruit development (60 days after full bloom), there were fewer volatile compounds, mainly aldehydes (87.0%). During fruit maturation (180 days after full bloom), the types and amounts of volatile compounds increased, mainly including esters (37.6%), and alkenes (23.2%). The total volatile concentration, the types of major volatile compounds, and their relative content in both cultivars varied across different stages. Gene expression analysis indicated that the upregulation of *MdLOX*, *MdAAT2*, and *MdADH3* was associated with increased aroma compound content, especially esters, during fruit development in both cultivars. Changes in the expression of *MdArAT*, *MdACPD*, *MdADH3*, *MdAAT2*, and *MdLOX* may lead to differences in volatile compounds between apple cultivars.

## 1. Introduction

Apple (*Malus domestica* Borkh.) is one of the most popular fruits worldwide, renowned for nutritional and taste quality, and high economic value. The quality of apple fruit is valued by consumers. The process of apple fruit development is regulated by multiple biochemical factors that affect the color, texture, flavor, and nutrition of fruit [1,2]. Aromatic volatile compounds are among the key factors that determine the fruit quality [3]. To date, more than 300 volatile compounds have been reported in apple fruit [4], mainly including aldehydes, esters, alcohols, alkenes, and ketones. Evidence suggests that the greatest contribution to apple sweetness is made by several volatile compounds, primarily esters [5].

Depending on the precursor required for the synthetic pathway, volatile compounds in apple fruit are synthesized via three main pathways; (i) biosynthesis of fatty acid-derived compounds, (ii) biosynthesis of amino acid derivatives, and (iii) biosynthesis of terpenoids [6,7]. The formation of volatile compounds in apple fruit is a dynamic process. Most apples are dominated by aldehydes in the early stages of fruit development, while esters become predominant during the mature stage [8,9,10]. In addition, there are differences in the identity, composition and concentration of volatile compounds between apple cultivars [11]. For example, the characteristic aroma of “Fuji” apple is mainly attributed to 2-methyl-1-butyl acetate, hexyl acetate, 2-methyl-butyric acid hexyl ester, 2-enyl hexyl ester, hexanal, 6-methyl-5-heptane En-2-one, and 2-hexenal, while the predominant volatile compounds in “Golden Delicious” apple are propyl acetate, butyl butyrate, *n*-butyl propionate, and butyl butyrate [12,13].

Recent studies have shown that some functional genes play potential roles in fruit aroma synthesis. Genes encoding enzymes with alcohol transaminase (AAT) activity have been isolated and characterized in various fruit species including strawberry, banana, and apple [14]. The *MdAAT1* gene has been shown to be involved in the production of fruit volatile esters such as hexyl acetate, butyl acetate, and 2-methylbutyl acetate found in “Royal Gala” apple [15]. In addition, alcohol dehydrogenase (ADH), which converts aldehydes of the lipoxygenase (LOX) pathway to alcohols, is one of factors regulating the production of volatile compounds during fruit maturation [16]. In tomato fruit, the catabolism of L-phenylalanine to aroma volatiles is initially mediated by aromatic amino acid decarboxylase (AADC) [17]. Moreover, aromatic amino acid aminotransferase (ArAT) and branched-chain amino acid transaminase (BCAT) are involved in the initial steps of amino acid-derived volatile synthesis in fruit [18,19].

Previous research on apple fruit aroma has mainly focused on the characterization of volatile profiles in specific cultivars. However, the molecular mechanisms underpinning the differences in volatile compounds produced by various cultivars, and the key factors affecting the synthesis of volatile compounds in apple, remain unclear. The “Fuji” cultivar is the most widely consumed throughout the world, while the recent “Ruixue” cultivar bred by crossing “Pink Lady” and “Fuji” cultivars has a unique flavor. The commercial harvest stage of “Ruixue” is ~5 days later than that of “Fuji”. Herein, we selected “Ruixue” and “Fuji” apples with considerably different flavors to explore changes in the volatile compounds and expression patterns of aroma-related genes during fruit development. In particular, we clarified the material basis and basic characteristics of the formation of volatile compounds in apple fruit. This work could provide useful data for the quality evaluation of apple fruit based on the biosynthesis of volatile compounds.

## 2. Results

### 2.1. Soluble Solids and Acidity of Apple Fruit during Different Stages

**Figure 1:**
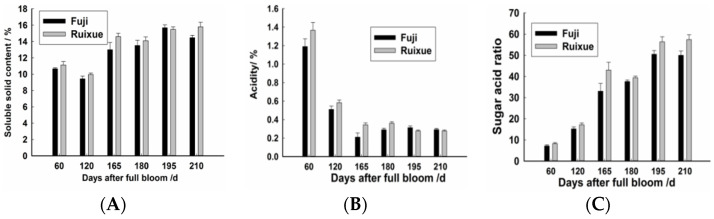
Quality parameters of “Ruixue” and “Fuji” apples during different stages of fruit development. (**A**) Soluble solid content; (**B**) Acidity; and (**C**) Sugar-acid ratio. Values are means ± standard error of three experimental replicates.

Changes in the quality parameters of the two apple cultivars are shown in Figure 1. The soluble solid content and sugar:acid ratio gradually increased with the development of fruit, while acidity decreased with increasing maturity. In “Ruixue”, the soluble solid content was 10.6% at 60 DAFB, while the mature fruit had a higher soluble solid content of 15.8% at 210 DAFB. The corresponding acidity decreased from 1.3% to 0.2%, while the sugar:acid ratio increased from 10.89 to 59.60. In “Fuji” apple, the soluble solid content was 9.4% at 60 DAFB, and this increased to 15.6% at 210 DAFB. The corresponding acidity decreased from 1.1% to 0.3%, while the sugar:acid ratio increased from 8.07 to 69.81. Notably, the soluble solid content of both cultivars increased dramatically from 120 to 165 DAFB, and their acidity levels decreased rapidly from 60 to 165 DAFB.

### 2.2. Volatile Compounds in Apple Fruit during Different Stages

**Figure 2:**
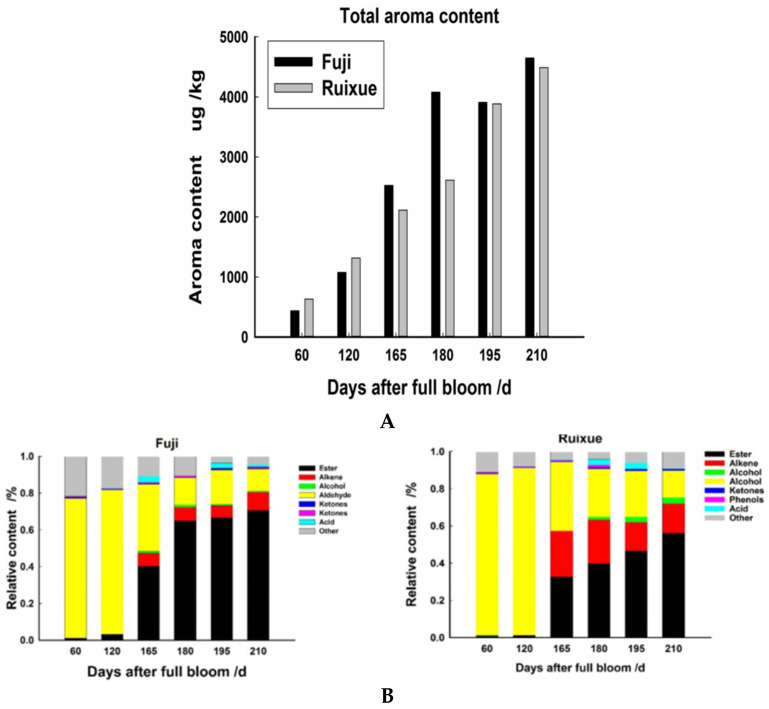

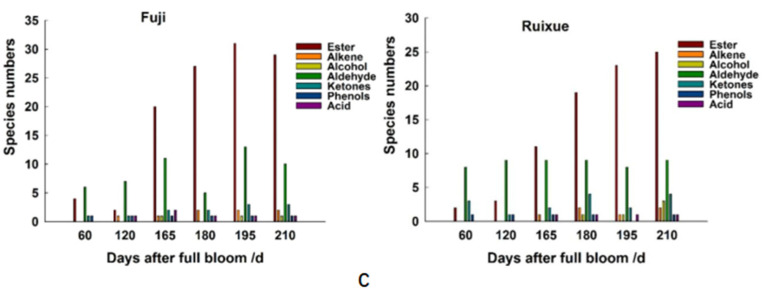
Volatile compounds in “Ruixue” and “Fuji” apples at different stages of fruit development. (**A**) Total volatile content; (**B**) Volatile compound relative content; (**C**) Volatile compound species numbers.

In “Ruixue” apple, the total volatile concentration continued to increase as fruit matured, from 633.50 μg/kg at 60 DAFB to 4488.42 μg/kg at 210 DAFB (Figure 2A). A total of 65 volatile compounds were detected in this cultivar, including 15 aldehydes, 34 esters, 2 acids, 5 ketones, 3 alcohols, 3 alkenes, and 3 other substances (Table 1). The major compounds were identified as 2-hexenal (64.89%), hexanal (13.30%), and 3-hexenal (6.08%) at 60 DAFB, while α-farnesene (22.99%), 2-hexenal (17.47%) and hexyl isovalerate (15.66%) were predominant at 180 DAFB. The relative content of volatile compounds changed dynamically with fruit development (Figure 2B). For example, the aldehyde content decreased, the ester content increased, while the alkene content increased first and then decreased with increasing maturity. The composition of esters changed the most, from two types at 60 DAFB to 25 types at 210 DAFB.

In “Fuji” apple, the increasing trend in total volatile concentration was essentially similar to that observed in “Ruixue” apple (Figure 2A). The total volatile concentration increased from 437.60 μg/kg at 60 DAFB to 4649.41 μg/kg at 210 DAFB, except for a decrease of 168.79 μg/kg at 195 DAFB relative to 180 DAFB. A total of 65 volatile compounds were detected in “Fuji” apple, including 15 aldehydes, 37 esters, 2 acids, 3 ketones, 2 alcohols, 3 alkenes, and 3 other substances (Table 2). The major volatile compounds were 2-hexenal (40.76%), hexanal (16.51%), and 3-hexenal (13.94%) at 60 DAFB. At 180 DAFB, the major compounds were hexyl isovalerate (15.73%), α-farnesene (11.37%), and 2-hexenal (11.02%). At 210 DAFB, the major compounds were hexenyl isovalerate (12.48%), hexyl hexanoate (12.42%), and α-farnesene (9.89%). During fruit development, the aldehyde content continued to decrease, while the ester content constantly increased. In addition, the alkene content increased from 0.1% at 120 DAFB to 10.0% at 210 DAFB (Figure 2B).

### 2.3. Expression of Genes Related to Aroma Synthesis during Different Stages

**Figure 3:**
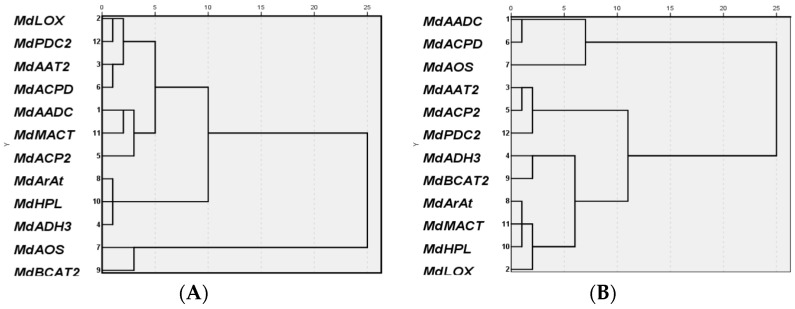
Cluster analysis of relative expression patterns of 12 genes related to the synthesis of volatile compounds in (**A**) “Ruixue” and (**B**) “Fuji” apple fruit.

**Figure 4:**
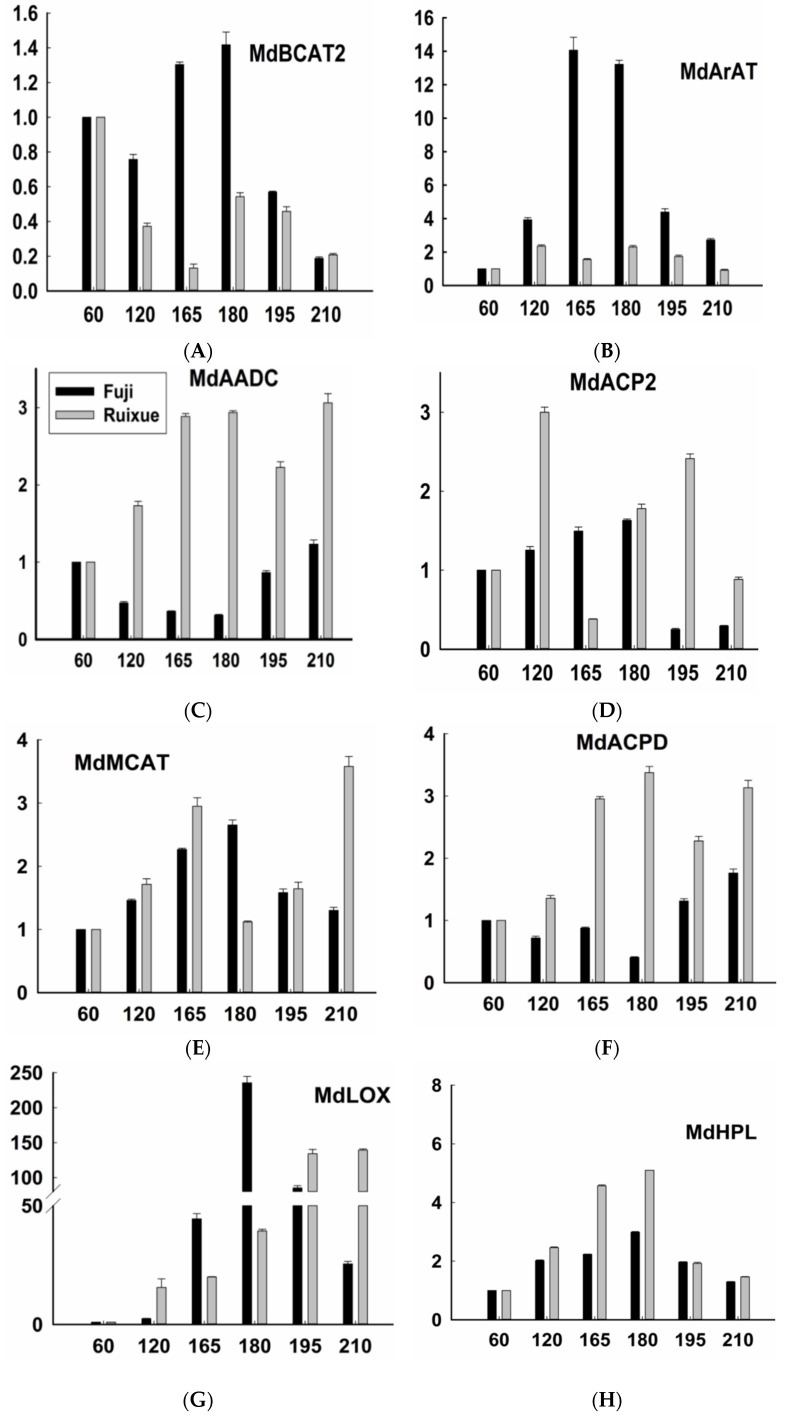

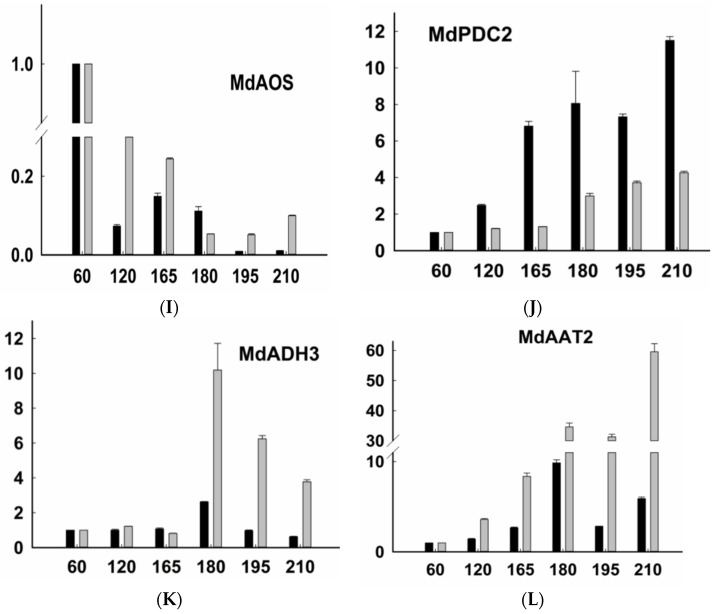
Changes in relative expression levels of 12 genes (**A**–**L**) related to aroma synthesis in fruit of “Ruixue” and “Fuji” apples during different developmental stages. Values are means ± standard error of three biological replicates.

Based on RT-qPCR data, the expression patterns of 12 candidate genes related to aroma synthesis in “Ruixue” apple could be divided into three classes (Figure 3). Class I comprised seven genes (*MdAADC*, *MdMCAT*, *MdLOX*, *MdACP2*, *MdACPD*, *MdPDC2*, and *MdAAT2*) and their relative expression levels increased with fruit development. Class II comprised three genes (*MdArAt*, *MdADH3*, and *MdADH3*) and their relative expression levels increased first then decreased with fruit development. Class III comprised two genes (*MdMACT* and *MdAOS*) whose relative expression levels continuously decreased with fruit development (Figure 4).

Expression patterns of the 12 genes in “Fuji” apple could also be divided into three classes (Figure 3). Class I comprised six genes (*MdBCAT2*, *MdArAT*, *MdMCAT*, *MdLOX*, *MdHPL* and *MdADH3*), for which relative expression levels increased in the early stages then decreased in the later stages of fruit development. There were three genes in class II (*MdAAT2*, *MdACP2*, and *MdPDC2*) with relative expression levels that first increased with fruit development, then decreased by 195 DAFB, then increased again by 210 DAFB. Class III also included three genes (*MdAADC*, *MdACPD*, and *MdAOS*) whose relative expression levels decreased first then increased with fruit development (Figure 4).

The results showed that variation in gene expression levels between “Ruixue” and “Fuji” apples during fruit development was related to differences in aroma synthesis in the two apple cultivars (Figure 4). Even when the gene expression patterns were the same, variation in gene expression levels was evident. This difference may be due to (i) variation in the overall expression level of each gene, and (ii) variation in the expression level of each gene over a certain time period. In “Ruixue” apple, the relative expression level of the *MdLOX* gene changed dramatically with fruit development; the relative expression level was highest at 210 DAFB, 140-fold higher than at 60 DAFB (Figure 3 and Figure 4). The expression patterns of *MdAAT2* and *MdLOX* were similar. Other genes of class I were relatively mildly expressed, with an increasing trend at different stages. For example, *MdPDC2* displayed the highest relative expression level at 210 DAFB, 4-fold higher than that at 60 DAFB. This phenomenon was also observed for the expression patterns of genes in classes II and III in “Ruixue” apple. In addition, there were significant differences in the overall gene expression of genes in “Fuji” apple across different stages. The relative expression level of the *MdLOX* gene was highest at 180 DAFB, 250-fold higher than at 60 DAFB. However, the relative expression of the *MdArAt* gene was highest at 165 DAFB, 14-fold higher than at 60 DAFB.

Changes in the expression of each gene over a certain period included sudden increases or decreases, or remaining stable for periods. During the development of “Ruixue” fruit, relative expression of the *MdLOX* gene increased suddenly from 39.35 at 180 DAFB to 134.10 at 195 DAFB. Meanwhile, relative expression of the *MdAAT2* gene increased suddenly from 8.37 at 120 DAFB to 34.60 at 165 DAFB. Relative expression levels of the *MdADH3* gene were relatively stable between 1.00 and 1.07 at 60, 120, and 165 DAFB. The relative expression level of the *MdLOX* gene decreased suddenly from 235.50 at 180 DAFB to 85.28 at 195 DAFB.

There were differences in expression patterns of genes related to aroma synthesis at different stages in the two apple cultivars. Differences in the relative expression of the *MdArAT* gene in “Fuji” were more evident than in “Ruixue”. The relative expression level of *MdArAT* in “Fuji” was 13.21 at 180 DAFB and 60 DAFB, compared with only 2.29 in “Ruixue”. In addition, the relative expression level of *MdAADC* began to increase in “Ruixue” during the early stages of fruit development, while the increase occurred in “Fuji” during the middle and late stages.

## 3. Discussion

In this study, we observed that soluble solid content, sugar:acid ratio, and total volatile concentration all increased with fruit development, while acidity decreased gradually in both “Fuji” and “Ruixue” apple cultivars. Previous work showed that aromatic volatile compounds in apple fruit are closely associated with harvest date and sugar content [20].

Apple aroma is a complex mixture of many volatile compounds produced by fruit [21]. The types and contents of volatile aroma substances of various fruits are very different from each other, which are largely affected by the variety, environment, cultivation, post-harvest storage and other conditions, especially the harvest maturity and other internal and external factors [22]. Only a few volatile compounds contribute substantially to fruit aroma, and most are esters, alcohols, aldehydes, and alkenes [23]. The identity, concentration, and quantities of these volatile compounds in fruit differ among cultivars [13]. Moreover, 31 volatile aroma substances were detected in Fuji. The main characteristic aromas of Fuji apple are 2-methyl hexanoate acetate, 2-methyl hexanoic acid propionyl and hexanoic acid hexanoate [24]. The main ingredients in the aroma of Marshal apple are 2-methyl hexyl butyrate, hexanal and 2-Hexenal, (E)- [25]. The aroma components in Royal Gala apples indicate that its main components are 2-methyl butyl acetate, butyl acetate, hexyl acetate and butanol [26]. According to reports, the aromatic volatile compounds in apples can be affected by weather, geographic location and temperature [27,28,29]. Cultivation measures also have a certain impact on the formation of aromatic volatile compounds in fruits, including factors such as irrigation, fertilization, and bagging [9,30]. When apple fruits are immature, they are mainly composed of C6 aldehydes, alcohols and other "green-flavor" aroma compounds. As the fruit matures, the content of these aldehydes and alcohols gradually decreases, and the C6–C10 esters and linalool with fruity aromas increases, and reaches the maximum content in mature fruits [31]. The storage environment will affect the formation of aromatic volatile compounds in apples [32], and the degree of influence depends on the storage conditions and the length of the storage period [33]. Storage conditions with high CO_2_ concentration may inhibit the formation of aromatic volatile compounds by inhibiting tricarboxylic acid, the prerequisite sub-stance of some aromatic volatile compounds [34].

The contribution of each compound to the specific aroma of fruit depends on the activity of relevant enzymes, the specificity and availability of substrates, and the presence of other compounds [35]. Herein, we found that the major volatile compounds detected in mature “Ruixue” fruit were hexyl butyrate α-farnesene, 2-hexenal hexyl isovalerate, and *n*-butyl butyrate (180–210 DAFB). Meanwhile, the major volatile compounds in mature “Fuji” fruit were hexyl isovalerate, α-farnesene, 2-hexenal, hexyl hexanoate, and butanoic acid 3-methyl-pentyl ester (195–210 DAFB). These results indicate that variation in the composition and content of aroma compounds leads to differences in aroma flavor between the two apple cultivars.

Based on RT-qPCR data, we explored the possible mechanisms underpinning the differences in aromatic volatile compounds between apple cultivars and the key factors affecting aroma synthesis in apple fruit. Straight-chain volatile compounds reportedly depend, to a large extent, on the availability of fatty acid-derived precursors [36]. Therefore, as the first step in the ester biosynthetic pathway, LOXs play a key role in volatile production in fruit [37]. A study based on gene scanning confirmed that multiple members of the *LOX* gene family are involved in the synthesis of apple aroma [38]. Herein, we found that in “Ruixue” apple, the relative expression of the *MdLOX* gene changed drastically with fruit maturation, and a 140-fold increase was observed at 210 DAFB compared with that at 60 DAFB. In “Fuji” apple, relative expression of the *MdLOX* gene also changed substantially, and a 250-fold increase was observed at 180 DAFB compared with that at 60 DAFB. When *MdLOX* expression began to increase, the ester content also increased sharply in both “Ruixue” and “Fuji” apples. In addition, hyperoxide lyase (HPL) is involved in the ester biosynthetic pathway, and HPL silencing reduces the production of most C6 volatiles in potato leaves [39]. It has been reported that the ratio of *MdHPL* to *MdAOS* may affect the biosynthesis of apple aroma [37]. Our results from the present study also suggest that this relationship may exist in “Ruixue” and “Fuji” apples.

Pyruvate decarboxylase (PDC) and ADHs are involved in the conversion between aldehydes and alcohols. PDC has been demonstrated to play a vital role in regulating the supply of pyruvate, one of the precursors for the formation of ethyl esters [40]. In the present study, we found that *MdPDC2* expression levels increased significantly in “Ruixue” and “Fuji” apples with increasing maturity. In addition, our results indicate that the relative expression of *MdADH3* increased sharply in “Ruixue” apple at 180 DAFB compared with 60 DAFB, but this change was not seen in “Fuji” apple.

Plant ArAT acts on L-tyrosine and L-phenylalanine. This enzyme is responsible for the direct decarboxylation of phenylalanine and it promotes the formation of fruit flavor [18]. Herein, we found that the *MdArAT* gene was expressed differently in the two apple cultivars, and a clear increasing trend was observed in “Fuji” apple during fruit development. However, such a change was not evident in “Ruixue” apple, and only the expression level of *MdACPD* increased obviously markedly in this cultivar during fruit development. *LeAADC1* or *LeAADC2* overexpression in transgenic tomato plants results in up to a 10-fold increase in emissions of products from this pathway in fruit [19]. Herein, our quantitative results showed that relative expression of *MdAADC* increased with fruit development in “Ruixue” apple, while its expression in “Fuji” fruit first decreased then increased with increasing maturity. In addition, fatty acid biosynthesis is one of the key pathways for aroma biosynthesis in apple fruit. Acyl carrier proteins (ACPs) are key factors involved in the fatty acid biosynthetic pathway [41]. Variation in the relative expression of *MdACP2* was relatively large and irregular in “Ruixue” apples during fruit development, while its expression level was relatively stable in “Fuji” apple. This expression difference could be one of the reasons for the difference in fruit aroma between the two apple varieties.

AAT is a key enzyme involved in the biosynthesis of ester compounds. Its main function is related to the binding of the acid donor acyl-CoA to alcohol receptors in the final key step of ester biosynthesis [42]. Northern blot and immunoblot assays revealed that the transcription and translation of *MdAAT2* are positively correlated with AAT enzyme activity and ester production in apple, except in the later maturation stage. This suggests that *MdAAT2* is involved in the regulation of ester biosynthesis, and that post-translational modification may be involved in the regulation of AAT activity [43]. The results of the present study showed that with the increasing maturity of “Ruixue” and “Fuji” apples, expression levels of *MdAAT2* increased considerably, while the content and number of ester compounds also increased. This result is consistent with previous findings for “Golden Delicious” and “Granny Smith” apples [44].

## 4. Materials and Methods

### 4.1. Plant Materials

Two apple (*Malus domestica Borkh*.) cultivars, “Ruixue” and “Fuji”, were produced at the apple experimental station of the Northwest A&F University (Yangling, Shaanxi Province, China). The station lies within the continental monsoon zone (109°32′ E, and 35°12′ N) in Baishui County, Shaanxi Province, China. The elevation of the experimental site is 830 m, and it has an average annual temperature of 11.6 °C and average annual rainfall of 567.6 mm.

Eight-year-old apple trees were grafted onto M26 rootstock, with a planting density of 3 m × 1 m. All fruit trees were cultivated in the same site conditions, with consistent flower thinning, fruit thinning, and fertilizer and water management levels. Ten fruit samples were collected from five trees with uniform growth at different stages, namely 60, 120, 165, 180, 195, and 210 days after full bloom (DAFB). Fruit samples without pests and mechanical damage were selected and used for subsequent analyses. The fruits of the same replicate group were scraped with a peeler to scrape the skin about 1 mm thick from the carcass, mixed into a sample, quickly frozen in liquid nitrogen and stored at −80 °C for later use.

### 4.2. Determination of Soluble Solids and Acidity

Total soluble solids were determined using a digital hand-held refractometer (Pocket PAL-1; Atago, Tokyo, Japan). Acidity was measured using a fruit acidity meter (GMK-835F; G-WON Hitech, Korea). The sugar:acid ratio was obtained by dividing the soluble solid content by the acidity. Each measurement was repeated three times.

### 4.3. Extraction and Determination of Volatile Compounds

Headspace solid-phase microextraction was used to extract volatile compounds from fruit peel samples. A 5 g sample was accurately weighed into a 50 mL bottle containing 1 g of sodium chloride, a stirrer bar, and 10 µL of the internal standard 3-nonanone (0.04 µL mL^−1^). After sealing and capping with tin foil. We placed it on a magnetic stirring heating plate and balance for 10 min. Subsequently, Solid phase microextraction head was inserted into the headspace of the sample bottle that had been equilibrated. After 40 min of adsorption, the extraction head was inserted into a gas chromatography inlet for thermal desorption at 250 °C. After 2.5 min of desorption, the extraction head was removed, and volatile compounds in the extract were analyzed using a GC-MSQP-2010 gas chromatograph-mass spectrometer (ISQ & TRACE ISQ; Thermo Fisher Scientific, Wilmington, DE, USA).

Gas chromatography was performed on an HP-INNOW capillary column, with a column length of 60 m, an inner diameter of 0.25 mm, and a liquid film thickness of 0.25 μm. The carrier gas was high-purity helium at a flow rate of 1.0 mL min^−1^, and the inlet temperature was set to 230 °C. Samples were injected by a splitless injection technique. The heating program involved an initial temperature of 40 °C maintained for 3 min, followed by a raise to 150 °C at a rate of 5 °C/min, then an increase to 220 °C at a rate of 10 °C/min, and holding for 5 min.

Mass spectrometry was performed using electrospray ionization, with an electron bombardment energy of 70 eV, an ion source temperature of 240 °C, a transmission line temperature of 240 °C, and an initial acquisition period of 2 min. Mass spectra of unknown volatile compounds were searched and matched against the NIST05 mass spectral library (http://www.sisweb.com/nist (accessed on 7 February 2021)) and supplemented by manual analysis to confirm their identities. The quantification of volatile compounds was based on peak area normalization to obtain the relative mass percentage of each compound, and 3-nonanone was used as the internal standard.

### 4.4. Total RNA Extraction, cDNA Synthesis, and Real-time Quantitative PCR

Total RNA was extracted from fruit samples using the improved cetyltrimethyl ammonium bromide (CTAB) method, as described previously [45], with slight modifications. The concentration of LiCl increased to 12 Mm. Subsequent cDNA synthesis was then performed using a PrimeScript RT Reagent Kit with gDNA Eraser (Takara, Dalian, China) following the manufacturer’s instructions.

Relative expression levels of 12 candidate genes encoding enzymes involved in aroma synthesis (e.g., BCAT2, ArAT, AADC, LOX, ADH3, and AAT2) were analyzed by quantitative real-time PCR (RT-qPCR) with cDNA as a temperate. Gene-specific primers were designed using Primer 5.0 (Premier Biosoft International, Palo Alto, CA, USA) and all primer sequences are listed in Table 3. *MdActin* was selected as the constitutively expressed reference gene and a sample (specified with an arbitrary number of 1) was used as a calibrator for “Ruixue” apples at 60 DAFB.

qRT-PCR analysis was performed using ChamQTM SYBRR qPCR Master Mix (Vazyme Biotech., Nanjing, China) with a Step OnePlus Real-Time PCR System (Applied Biosystems, Foster, CA, USA). The cycling parameters were 95 °C for 30 s followed by 40 cycles at 95 °C for 5 s and 60 °C for 30 s. All reactions were performed in triplicate for each of the three biological replicates, and relative expression levels of the selected genes were calculated using the relative 2^−∆∆CT^ method [46].

### 4.5. Statistical Analysis

Experimental data are presented as means ± standard error of three experimental replicates. All data were processed using Excel 2010 (Microsoft Corp., Redmond, WA, USA). A one-way analysis of variance (ANOVA) was performed on data using IBM SPSS 21.0 (IBM Corp., Armonk, NY, USA). Graphs were drawn using SigmaPlot 12.5 (Systat Software Inc., San Jose, CA, USA).

## 5. Conclusions

This study showed that the major volatile compounds in apple were aldehydes during the early stage of fruit development (60–120 DAFB). In “Ruixue” and “Fuji” apples, they were mainly 2-hexenal, *n*-hexanal, and 3-hexenal. With increasing maturity (165–210 DAFB), the content of aldehydes decreased, while the content and types of other volatile compounds (especially esters and alkenes) gradually increased. In “Ruixue” apple, there were α-farnesene, hexyl isovalerate, butanoic acid, 3-methyl-, pentyl ester, and hexyl hexanoate. In “Fuji” apple, there were mainly α-farnesene, hexyl isovalerate, hexyl butyrate, and butyl butyrate. Gene expression analysis revealed that increased expression of *MdLOX*, *MdAAT2*, and *MdADH3* was associated with increased aroma compound content, especially esters, in “Ruixue” and “Fuji” apples during fruit development. Changes in the expression of *MdArAT*, *MdACPD*, *MdADH3*, *MdAAT2*, and *MdLOX* may have contributed to differences in volatile compounds between the two apple cultivars.

Based on the above results, the following can be concluded:

In the early stage of apple fruit development (60–120 DAFB), most of the volatile compounds in fruits are aldehydes. In “Ruixue” apples and “Fuji” apples, they are mainly 2-hexenal, *n*-hexanal, and 3-hexenal. When the maturity increases, the content of aldehydes decreases, while the content and types of other volatile compounds (especially esters and alkenes) gradually increase. In “Ruixue” apples, there are α-farnesene, hexyl isovalerate, butanoic acid, 3-methyl-, pentyl ester and hexyl hexanoate. In “Fuji” apples, there are mainly α-farnesene, hexyl isovalerate, hexyl butyrate and butyl butyrate.

Our comprehensive study demonstrates that increased expression of *MdLOX*, *MdAAT2*, and *MdADH3* is associated with increased aroma, especially esters, in “Ruixue” and “Fuji” apples during fruit development. Changes in the expression of *MdArAT*, *MdACPD*, *MdADH3*, *MdAAT2*, and *MdLOX* may lead to differences in volatile compounds between the two apple cultivars.

## Figures and Tables

**Table 1:** Major volatile compounds detected in “Ruixue” apple fruit during different developmental stages (DAFB, days after full bloom).

No.	Compound	Relative Content/%					
		60 DAFB	120 DAFB	165 DAFB	180 DAFB	195 DAFB	210 DAFB
	**Aldehydes**						
1	Hexanal	13.3 ± 0.64	11.67 ± 0.54	3.63 ± 0.31	2.51 ± 0.17	2.01 ± 0.21	2.34 ± 0.05
2	3-Hexenal	6.08 ± 0.34	10.03 ± 0.56	2.19 ± 0.25	2.57 ± 0.22	0.35 ± 0.08	0.92 ± 0.04
3	2-Hexenal	64.89 ± 4.37	63.31 ± 5.14	25.25 ± 2.35	17.47 ± 1.32	14.85 ± 0.98	14 ± 1.24
4	(*E*)-2-Octenal	0.08 ± 0.01	-	0.35 ± 0.07	0.3 ± 0.02	0.38 ± 0.04	0.31 ± 0.12
5	Dodecanal	-	-	0.2 ± 0.06	-	0.26 ± 0.02	-
6	(*E*)-2-Hexenal	-	-	2.18 ± 0.12	-	-	0.97 ± 0.17
7	5-Hydroxymethylfurfural	-	-	-	-	3.97 ± 0.17	-
8	Octanal	-	0.15 ± 0.04	-	0.37 ± 0.07	0.34 ± 0.06	0.39 ± 0.04
9	Nonanal	0.4 ± 0.08	0.57 ± 0.07	0.54 ± 0.12	0.71 ± 0.06	-	0.65 ± 0.07
10	(*Z*)-2-Heptenal	0.13 ± 0.01	0.2 ± 0.08	0.42 ± 0.14	0.55 ± 0.10	-	0.6 ± 0.04
11	Decanal	0.32 ± 0.13	0.46 ± 0.11	-	0.11 ± 0.02	-	0.22 ± 0.02
12	(*E*)-2-Decenal	-	-	-	0.05 ± 0.01	-	-
13	(*E,E*)-2,4-Hexadienal	1.32 ± 0.18	2.64 ± 0.13	0.32 ± 0.04	-	-	-
14	Furfural	-	-	-	-	1.21 ± 0.07	-
15	2-Methyl-4-pentenal	-	0.27 ± 0.02	-	-	-	-
	**Esters**						
16	Alkyl isobutyrate	-	-	-	2.57 ± 0.39	-	-
17	Butyl 2-methylbutanoate	-	-	-	-	1.82 ± 0.23	-
18	Isoamyl butyrate	-	-	-	1.26 ± 0.35	1.23 ± 0.29	1.64 ± 0.49
19	Hexyl acetate	0.09 ± 0.02	0.64 ± 0.17	2.14 ± 0.21	0.98 ± 0.14	0.34 ± 0.01	0.23 ± 0.06
20	Hexyl propionate	-	-	0.95 ± 0.19	2.58 ± 0.29	2.89 ± 0.32	3.05 ± 0.28
21	Hexyl isovalerate	-	-	20.89 ± 2.31	15.66 ± 2.38	11.15 ± 0.55	17.57 ± 1.64
22	Octaethylene glycol Monododecyl ether	-	0.42 ± 0.03	0.62 ± 0.15	0.10 ± 0.01	0.22 ± 0.04	0.31 ± 0.02
23	Butanoic acid, 2-methyl-, pentyl ester	-	-	0.9 ± 0.19	1.32 ± 0.19	1.46 ± 0.12	1.7 ± 0.16
24	Butanoic acid, butyl ester	-	-	0.39 ± 0.07	0.96 ± 0.05	2.26 ± 0.25	10.91 ± 1.24
25	2-Methylbutyl 2-Methylbutanoate	-	-	0.61 ± 0.25	2.03 ± 0.35	1.74 ± 0.24	3.05 ± 0.29
26	Hexanoic acid, hexyl ester	-	-	1.39 ± 0.14	-	4.2 ± 0.43	7.53 ± 0.56
27	Octanoic acid, hexyl ester	-	0.06 ± 0.01	-	-	-	0.19 ± 0.02
28	Butanoic acid, pentyl ester	-	-	0.23 ± 0.06	0.61 ± 0.19	0.68 ± 0.12	1.51 ± 0.35
29	2-Methylbutanoic acid-2-methylbutyl ester	-	-	0.17 ± 0.04	-	-	-
30	Propanoic acid, butyl ester	-	-	-	0.37 ± 0.14	0.36 ± 0.03	0.41 ± 0.03
31	Octylhexanoate	-	-	-	-	-	-
32	2-Hexen-1-ol, acetate	0.43 ± 0.14	-	-	-	-	-
33	Hexyl butyrate	-	-	-	5.06 ± 0.38	16.65 ± 1.23	10.91 ± 1.05
34	Hexanoic acid, 2-methylbutyl ester	-	-	-	1.84 ± 0.32	2.05 ± 0.22	3.05 ± 0.21
35	Hexanoic acid, pentyl ester	-	-	-	0.89 ± 0.05	-	-
36	Propyl octanoate	-	-	-	-	0.04 ± 0.01	0.05 ± 0.01
37	Heptyl 2-methylbutyrate	-	-	-	0.23 ± 0.01	0.28 ± 0.04	0.24 ± 0.03
38	2-Methylbutyl octanoate	-	-	-	-	-	0.37 ± 0.04
39	Heptanoic acid, butyl ester	-	-	-	-	-	0.22 ± 0.02
40	Butanoic acid, 2-methyl-, propyl ester	-	-	-	0.25 ± 0.05	0.21 ± 0.19	0.18 ± 0.03
41	Propyl hexanoate	-	-	-	-	0.34 ± 0.27	-
42	Ethyl butyrate	-	-	-	-	-	0.17 ± 0.02
43	Propanoic acid, pentyl ester	-	-	-	0.66 ± 0.14	0.43 ± 0.06	0.63 ± 0.04
44	Hexanoic acid, 2-methylpropyl ester	-	-	-	-	0.08 ± 0.01	0.1 ± 0.01
45	Pentanoic acid, 2-methylbutyl ester	-	-	-	0.13 ± 0.02	0.22 ± 0.19	0.16 ± 0.01
46	Propanoic acid, propyl ester	-	-	-	-	0.12 ± 0.02	-
47	Isopentyl hexanoate	-	-	0.29 ± 0.04	-	-	-
48	(*Z,Z,Z*)-9,12,15-Octadecatrienoic acid, 2,3-dihydroxypropyl ester	-	-	-	0.09 ± 0.01	-	-
49	Butanoic acid, propyl ester	-	-	-	-	-	0.32 ± 0.05
	**Acids**						
50	2-Methyl-mutanoic acid	-	-	0.35 ± 0.03	3.02 ± 0.02	1.76 ± 0.12	0.16 ± 0.02
51	Acetic acid	-	-	-	-	1.47 ± 0.13	-
	**Ketones**						
52	1-Octen-3-one	0.07 ± 0.01	-	0.25 ± 0.03	0.32 ± 0.03	0.42 ± 0.03	0.38 ± 0.04
53	6-Methyl-5-hepten-2-one	0.35 ± 0.03	0.45 ± 0.06	0.24 ± 0.02	0.36 ± 0.02	0.45 ± 0.02	0.27 ± 0.03
54	(*E*)-6,10-Dimethyl-,5,9-undecadien-2-one	-	-	-	0.07 ± 0.01	-	0.11 ± 0.01
55	4,4-Dimethyl-3-phenyl-2,5-cyclohexadien-1-one	-	-	-	-	-	0.23 ± 0.03
56	Acetone	0.1 ± 0.01	-	-	0.05 ± 0.01	-	-
	**Alcohols**						
57	1-Hexanol	-	-	-	1.39 ± 0.15	-	1.45 ± 0.20
58	1-Butanol, 2-methyl-, (*S*)-	-	-	-	-	1.21 ± 0.14	1.04 ± 0.17
59	1-Undecanol	-	-	-	-	-	0.14 ± 0.02
	**Alkene**						
60	(*E*)-á-Famesene	-	-	-	0.19 ± 0.02	-	-
61	à-Farnesene	-	-	27.99 ± 1.96	22.99 ± 1.65	15.22 ± 1.23	15.47 ± 1.35
	**Others**						
62	Estragole	-	-	0.11 ± 0.01	-	-	0.08 ± 0.01
63	1-Nonene	-	-	-	-	-	0.18 ± 0.02
64	Melezitose	-	-	-	-	0.48 ± 0.03	-
65	2,4-Di-tert-butylphenol	0.63 ± 0.05	0.34 ± 0.02	0.51 ± 0.04	1.44 ± 0.12	-	0.09 ± 0.01

Note: Values are means ± standard error of three biological replicates; -, no such substance has been detected.

**Table 2:** Major volatile compounds detected in “Fuji” apple fruit during different developmental stages (DAFB, days after full bloom).

No.	Compound	Relative Content/%					
		60 DAFB	120 DAFB	165 DAFB	180 DAFB	195 DAFB	210 DAFB
	**Aldehydes**						
1	Hexanal	16.51 ± 0.7	16.4 ± 0.31	3.27 ± 0.18	1.84 ± 0.27	1.26 ± 0.23	1.3 ± 0.07
2	3-Hexenal	13.94 ± 0.46	10.67 ± 0.48	5.49 ± 0.98	0.86 ± 0.16	0.12 ± 0.02	0.47 ± 0.03
3	2-Hexenal	40.76 ± 0.22	45.86 ± 0.49	19.9 ± 1.13	11.02 ± 0.82	11.5 ± 0.85	8.33 ± 0.68
4	(*E*)-2-Octenal	-	-	0.37 ±	0.2 ± 0.05	0.6 ± 0.14	0.21 ± 0.05
5	Dodecanal	-	-	-	-	0.09 ± 0.19	-
6	(*E*)-2-Hexenal	-	-	4.06 ± 0.77	-	2.58 ± 0.34	-
7	Octanal	-	-	0.28 ± 0.03	-	0.38 ± 0.24	0.25 ± 0.04
8	Nonanal	-	-	-	-	0.87 ± 0.05	0.56 ± 0.13
9	(*Z*)-2-Heptenal	-	0.31 ± 0.04	0.69 ± 0.07	-	0.58 ± 0.17	0.33 ± 0.05
10	(*Z*)-2-Nonenal	-	-	-	0.09 ± 0.01	0.14 ± 0.04	0.14 ± 0.01
11	Decanal	0.4 ± 0.02	0.51 ± 0.03	-	-	-	-
12	(*E*)-2-Decenal	-	-	0.07 ± 0.01	-	0.12 ± 0.07	0.1 ± 0.01
13	(*E,E*)-2,4-Hexadienal	4.83 ± 0.51	6.11 ± 0.15	0.24 ± 0.13	-	-	-
14	Furfural	-	-	2.07 ± 0.23	-	0.77 ± 0.22	-
15	2-Methyl-4-Pentenal	0.52 ± 0.23	0.19 ± 0.05	0.45 ± 0.17	-	0.08 ± 0.02	0.03 ± 0.01
	**Esters**						
16	Butyl 2-methylbutanoate	-	-	-	3.39 ± 0.35	2.7 ± 0.04	1.97 ± 0.24
17	Isoamyl butyrate	-	-	0.62 ± 0.04	0.75 ± 0.19	0.57 ± 0.12	0.74 ± 0.04
18	Hexyl acetate	0.34 ± 0.02	0.93 ± 0.08	4.79 ± 0.58	4.85 ± 0.46	6.13 ± 0.23	4.02 ± 0.34
19	Hexyl propionate	-	-	0.79 ± 0.15	2.8 ± 0.35	2.98 ± 0.40	2.45 ± 0.25
20	Hexyl isovalerate	-	-	10.78 ± 0.77	15.73 ± 0.87	14.16 ± 0.55	12.48 ± 0.64
21	Octaethylene glycol monododecyl ether	0.86 ± 0.21	2.06 ± 0.70	0.68 ± 0.04	-	0.34 ± 0.31	-
22	Acetic acid, butyl ester	-	-	0.94 ± 009	1.33 ± 0.24	1.67 ± 0.17	1.65 ± 0.33
23	Butanoic acid, 2-methyl-, pentyl ester	-	-	0.93 ± 0.12	1.29 ± 0.04	0.94 ± 0.16	1.39 ± 0.14
24	Butanoic acid, butyl ester	-	-	1.19 ± 0.22	1.68 ± 0.34	2.46 ± 0.23	2.05 ± 0.30
25	Butyl 2-methylbutanoate	-	-	1.74 ± 0.07	-	1.56 ± 0.18	1.72 ± 0.18
26	Hexyl Hexanoate	-	-	3.83 ± 0.18	-	5.55 ± 0.37	12.42 ± 0.47
27	Hexyl octanoate	-	-	-	0.42 ± 0.26	0.23 ± 0.04	0.37 ± 0.02
28	1-Butanol, 2-methyl-, acetate	-	-	4.59 ± 0.97	4.2 ± 0.62	2.09 ± 0.17	5.58 ± 0.34
29	Propanoic acid, butyl ester	-	-	-	1.22 ± 0.24	0.27 ± 0.09	1.64 ± 0.04
30	Acetic acid, pentyl ester	-	-	-	0.45 ± 0.07	0.09 ± 0.01	0.91 ± 0.09
31	Octylhexanoate	-	-	0.17 ± 0.03	-	0.19 ± 0.03	-
32	Hexanoic acid, 2-methylbutyl ester	-	-	1.96 ± 0.04	2.54 ± 0.33	1.79 ± 0.23	2.95 ± 0.24
33	Hexanoic acid, pentyl ester	-	-	0.92 ± 0.01	-	-	-
34	Propyl octanoate	-	-	0.08 ± 0.01	0.37 ± 0.18	0.71 ± 0.21	0.55 ± 0.04
35	2-Methylbutyl 2-methylbutanoate	-	-	-	0.3 ± 0.10	0.22 ± 0.04	0.27 ± 0.03
36	Butyl caprylate	-	-	-	1.96 ± 0.09	2.03 ± 0.31	-
37	2-Methylbutyl octanoate	-	-	-	1.15 ± 0.24	0.72 ± 0.14	0.92 ± 0.04
38	Heptanoic acid, butyl ester	-	-	-	0.28 ± 0.08	2.18 ± 0.22	0.58 ± 0.16
39	Butyl caprate	-	-	-	0.05 ± 0.01	0.05 ± 0.01	0.04 ± 0.1
40	*n*-Propyl acetate	-	-	-	-	-	0.28 ± 0.18
41	Heptyl 2-methylbutyrate	-	-	-	0.65 ± 0.21	0.14 ± 0.01	1.28 ± 0.007
42	Propyl acetate	-	-	0.22 ± 0.07	0.96 ± 0.17	0.84 ± 0.03	1.93 ± 0.16
43	Butanoic acid3-methyl-, pentyl ester	-	-	5.03 ± 0.53	-	10.66 ± 0.56	8.14 ± 0.29
44	Acetic acid, heptyl ester	-	-	-	0.04 ± 0.01	0.04 ± 0.01	0.03 ± 0.01
45	Acetic acid, 2-phenylethyl ester	0.12 ± 0.01	-	-	-	-	-
46	Propanoic acid, pentyl ester	-	-	-	0.71 ± 0.13	1.16 ± 0.07	1.57 ± 0.26
47	Hexanoic acid, 2-methylpropyl ester	-	-	0.13 ± 0.01	0.08 ± 0.01	0.1 ± 0.01	0.1 ± 0.04
48	Pentanoic acid, 2-methylbutyl ester	-	-	-	0.08 ± 0.01	0.05 ± 0.01	0.13 ± 0.06
49	Propanoic acid, propyl ester	-	-	-	0.22 ± 0.05	0.21 ± 0.03	0.3 ± 0.18
50	Octanoic acid, 2-ethylhexyl ester	-	-	0.36 ± 0.07	-	-	-
51	Cis-3-Hexenyl isovalerate	-	-	-	0.06 ± 0.01	-	-
52	Octanoic acid, 2-ethylhexyl ester	-	-	0.36 ± 0.07	-	-	-
53	Heptyl 2-methylbutyrate	-	-	0.88 ± 0.02	-	-	-
	**Acids**						
54	2-Methyl-butanoic acid	-	-	1.35 ± 0.18	2.81 ± 0.42	1.85 ± 0.22	1.28 ± 0.21
	**Ketones**						
55	1-Octen-3-one	-	-	0.23 ± 0.04	0.13 ± 0.20	0.3 ± 0.02	0.21 ± 0.02
56	6-Methyl-5-hepten-2-one	0.37 ± 0.01	0.61 ± 0.10	0.17 ± 0.02	-	0.26 ± 0.02	0.2 ± 0.04
57	(*E*)-6,10-Dimethyl-,5,9-undecadien-2-one	-	-	-	0.11 ± 0.19	0.08 ± 0.01	0.06 ± 0.01
	**Alcohols**						
58	1-Hexanol	-	-	-	-	0.61 ± 0.34	0.6 ± 0.01
59	(*S*)-2-Methyl-1-butanol	-	-	0.41 ± 0.05	-	-	-
	**Alkene**						
60	(*E*)-á-Famesene	-	-	-	0.04 ± 0.01	-	0.13 ± 0.02
61	à-Farnesene	-	0.12 ± 0.01	16.44 ± 1.49	11.37 ± 1.12	6.65 ± 0.71	9.88 ± 0.54
62	1-Nonene	-	-	-	-	0.11 ± 0.04	-
	**Others**						
63	Estragole	-	-	-	0.04 ± 0.01	-	0.1 ± 0.01
64	2,4-Di-tert-butylphenol	0.56 ± 0.02	0.41 ± 0.03	0.63 ± 0.17	0.8 ± 0.12	0.39 ± 0.06	0.75 ± 0.03
65	Carbamic acid, monoammonium salt	-	1.26 ± 0.13	-	-	-	-

Note: Values are means ± standard error of three biological replicates; -, no such substance has been detected.

**Table 3:** Genes and primers used for quantitative real-time PCR.

Gene Name	Protein Product	Forward Primer Sequence (5′-3′)	Reverse Primer Sequence (5′-3′)	GenBank Accession No.
*MdBCAT2*	Branched-chain amino acid aminotransferase 2	ACTCGTTCCTCAACCTGGTA	AAGCAAGGGATTTTCGGATGTT	CV082955
*MdArAT*	Aromatic amino acid aminotransferase	CAGTCGTCACAAACCAACC	GCTGATGAAGTTTATGGGCA	GO511145
*MdAADC*	Amino acid decarboxylase	GTTGATGATGGCGGGTTTATC	AGGCAATCTTCACGGAGTCTT	CN888142
*MdACP2*	Acyl carrier protein 2	CCGAGTACATAACCAGTCTACCA	CGCAGATGTTATTGAGAAGCTC	CV630926
*MdMCAT*	Malonyl-CoA:ACP transacylase	AGGCCGCACGTGACATCAAC	GACGCCTTGTTCGCCGATTAC	MD177270
*MdACPD*	Acyl-ACP desaturase	CTCAGCCCAATCCTCTAATGACTT	CTCCACTCTTCACTCCTCCACC	ES790074
*MdLOX*	Lipoxygenase	GTCACGCTGTCCGAGATAGA	ACTCCAGAGTATGAGGAGCTCAG	DY742295
*MdHPL*	Hydroperoxide lyase	GTGTGAACTGAGTTGGAAGTCCT	TTGCAACTGGTTCAGTCAGTAGT	GO537614
*MdAOS*	Alene oxide synthase	CGACTCGACTTGAGGAGGTAG	AAGAAGGATATCTTCACCGGAAC	DR991430
*MdPDC2*	Pyruvate decarboxylase 2	TGGTCAGTTGGAGCAACTCTT	GACACATCTTGAGCAGTCACCT	DR992977
*MdADH3*	Alcohol dehydrogenase 3	CCGTCGATCATGAGATTATCC	AACTTGCAAGGAGTGGAACTG	DY256060
*MdAAT2*	Alcohol acyltransferase 2	AGGACAACCAATAATTCCATCAG	GATGTCACACTTGAGCAACTAGG	AY517491
*MdActin* (reference gene)		GCCAGATCTTCTCCATGTCATCC	TGTGTTTCCTAGTATTGTTGGTCGC	XM008393049

## Data Availability

The data used to support the findings of this study are available from the corresponding author upon request.

## Sample Availability

Samples of two apple cultivars material, “Ruixue” and “Fuji” are available from the authors.
